# Coffee consumption has no acute effects on glucose metabolism in healthy men: a randomized crossover clinical trial

**DOI:** 10.1186/1758-5996-7-S1-A224

**Published:** 2015-11-11

**Authors:** Caio Eduardo Gonçalves Reis, Sara Wassell, Adriana L Porto, Angélica A Amato, Leslie JC Bluck, Teresa HM da Costa

**Affiliations:** 1 Universidade de Brasília, Guará, Brazil

## Background

Multiple epidemiologic studies have consistently reported association between increased coffee consumption and a lowered risk of Type 2 diabetes mellitus. However, the mechanisms behind this finding have not been fully elucidated.

## Objective

We investigate the effect of coffee (caffeinated and decaffeinated) on glucose effectiveness and insulin sensitivity using the stable isotope minimal model protocol with oral glucose administration in healthy men.

## Materials and methods

Fifteen healthy men underwent 5 arms randomized crossover single-blinding (researchers) clinical trial. They consumed decaffeinated coffee, caffeinated coffee (with and without sugar), and controls – water (with and without sugar) followed 1 hour by an oral glucose tolerance test (75 g of available carbohydrate) with intravenous labeled dosing interpreted by the two compartment minimal model (225 min). One-way ANOVA with Bonferroni adjustment were used to compare the effects of the tested beverages on glucose metabolism parameters.

## Results

Decaffeinated coffee resulted in 29% and 85% higher insulin sensitivity compared with caffeinated coffee and water, respectively, and the caffeinated coffee showed 15% and 60% higher glucose effectiveness compared with decaffeinated coffee and water, respectively. However, these differences were not significant (p >0.10). In overall analyze (0 – 225 min) there were no significant differences on glucose effectiveness, insulin sensitivity, and glucose and insulin area under the curve between the groups. The beneficial effects of coffee did not seem to act in the short-term (h) on glucose metabolism parameters mainly on insulin sensitivity indices. The benefits of coffee consumption occur in the long-term (yrs.) as has been shown in the reduction of Type 2 Diabetes Mellitus risk in epidemiological studies. The clinical relevance of the present findings is that there is no need to avoid coffee as the drink choice for healthy people.

## Conclusions

The findings of this study demonstrate that the consumption of caffeinated and decaffeinated coffee with or without sugar has no acute effects on glucose metabolism in healthy men. Further researches, including long-term interventional studies, are needed to fully elucidate the mechanisms behind the coffee effects on reduced risk for Type 2 diabetes mellitus.

